# In Vivo Evaluation of the Position and Orientation of the Geometric Axis of the Tibiotalar Joint

**DOI:** 10.1155/2023/2763099

**Published:** 2023-01-18

**Authors:** Jian Yu, Shengxuan Cao, Chen Wang, Dahang Zhao, Shuo Wang, Chao Zhang, Jiazhang Huang, Xu Wang, Xin Ma

**Affiliations:** ^1^Department of Orthopedics, Huashan Hospital, Fudan University, Shanghai, China; ^2^Department of Orthopedics, Ruijin Hospital, Shanghai Jiao Tong University School of Medicine, Shanghai, China

## Abstract

**Background:**

Fitting the surface morphology of the talar trochlea is one of the common methods to define the geometric axis of the tibiotalar joint (GATJ). However, the in vivo motion of such axis during gait has not been fully investigated.

**Methods:**

The ankle kinematic data of fifteen volunteers were collected by a dual fluoroscopic imaging system with a model-image registration method. The GATJ was defined by sphere-fitting the medial or lateral part of the trochlear surface of the talus. The position and orientation of this axis during gait were measured. To verify this axis, the distances of the feature points of the talus to the GATJ during gait were also measured.

**Results:**

There was no statistically significant difference in the distances of feature points of the talus to the GATJ among the seven key poses of the gait cycle. And the position and orientation of the GATJ during gait also showed no statistically significant difference.

**Conclusion:**

The GATJ is the axis about which the talus rotated. And it is one fixed axis during gait. The current finding may help the design of the talar component for total ankle replacement based on the surface morphology of the talar trochlea.

## 1. Introduction

Accurately defining the rotational axis of the tibiotalar joint is essential to the understanding of the ankle joint biomechanics and implant design for total ankle replacement. Total ankle implants considering a kinematic alignment orientated to the joint axis can better mimic the natural ankle motion and may reduce postoperative complications, such as impingement, thus improving the clinical results. However, the rotational axis has not been fully investigated and no consensus on the axis about which the talus rotates in the mortise has been established [1, 2]. The debate of one fixed axis, two distinct axes for plantar flexion and dorsiflexion, or multiple axes still remains [1–5].

The rotational axis of the tibiotalar joint was commonly determined either by kinematic data of the ankle joint [6–9] or by three-dimensional (3D) bone geometry of the talus [4, 5, 10]. During ankle motion, the tibial plafond is in contact with the talar trochlea, especially the anterior and posterior regions [11]. Therefore, the shape of the talar trochlea should determine the axis of rotation of the tibiotalar joint. However, geometric axes determined under a static condition may not be a reasonable representative of weight-bearing activities, such as walking. Whether the talus rotates about the geometric axis of the tibiotalar joint (GATJ) and how this axis changes during gait have not yet been investigated.

Due to the lack of accurate and reliable methods for experimentally determining the rotational axis, early attempts [2, 12] used specially designed apparatuses to directly measure the rotational axis on cadaveric specimens or in vivo. However, such methods can not accurately isolate the single joint motion. Several studies [7, 13] later in vivo tracked the motion of skin markers sticked to the feet of volunteers during walking or cadaveric specimens above a foot simulation machine with cameras. Then the rotational axis can be found with best fit kinematic models. Unfortunately, the talus, located in the middle of the ankle complex, cannot be tracked with skin-mounted markers in vivo. An alternative approach [6, 14] was to use metal pins implanted in volunteers' bones, which was highly invasive and not recommended.

Dual fluoroscopy technique with model-image registration method has proved to have an accuracy of about 0.1 mm in position and 0.1 degrees in orientation [15, 16]. This method enables us to obtain the spatial position of the tibial and talus during gait, thus better understanding the kinematic characteristics of the tibiotalar joint and its geometric rotational axis. The objective of this study was to use the dual fluoroscopy technique to investigate the in vivo changes of the GATJ.

## 2. Materials and Methods

### 2.1. Subjects

The experimental data used in this study have been previously described [17, 18]. In brief, fifteen healthy volunteers (8 females, 7 males; age, 26.2 ± 5.4 years; height, 170.4 ± 6.9cm; mass, 65.6 ± 14.0 kg) were recruited in this study. The study protocol was reviewed and approved by the ethics committee of the authors' institute (HIRB Approve Letter (2021) No. 457). All subjects provided informed consent before participation. The ankle of each subject was physically examined before enrollment. Subjects with pain, deformity, injury, or surgery history in either lower limb were excluded.

### 2.2. CT Scan and Ankle Modeling

The experimental protocol for data collection has also been outlined previously [17, 18]. Each involved ankle was computerized tomographic (CT) scanned (Light Speed; GE, Milwaukee, WI, USA) from 10 cm above tibial plafond to toe tip with 0.67 mm of thickness, 120 kV of voltage, and 200 mA of current. The images with Digital Imaging and Communications in Medicine (DICOM) file type were segmented and reconstructed into 3D models of the tibia and talus by MIMICS (Materialise NV. Leuven, Belgium).

### 2.3. Dual Fluoroscopy Imaging and Model Registration

**Figure 1:**
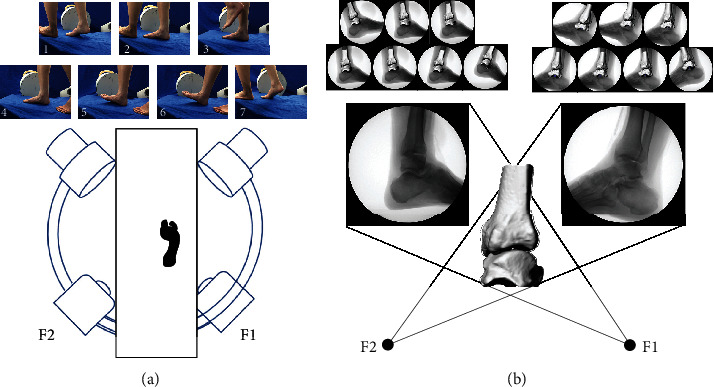
(a) Seven key poses of the right foot selected for analysis and illustration of the dual fluoroscopic system (two approximately orthogonal fluoroscopies F1 and F2). (b) Model-image registration method to reproduce the in vivo position of the bones of the hindfoot.

A previously validated [15] dual fluoroscopy imaging system was used to acquire the in vivo kinematics data. The dual fluoroscopy imaging system included two approximately orthogonal fluoroscopies (BV Pulsera, Phillips Medical, USA) with a resolution of 1024 × 1024 pixels and beam energy settings of 75 kV and 40 mA at a frame rate of 30 Hz and a custom-made walking platform. The subjects were instructed to walk slowly at approximately 1 m/s. The following seven key poses were selected to analyze the tibiotalar kinematics (Figure 1): (1) heel strike of the tested side; (2) foot flat of the tested side; (3) mid-stance of the tested side and toe-off of the other side; (4) mid-stance of the tested side and swing phase of the other side; (5) heel off of the tested side and preparation for heel strike of the other side; (6) heel strike of the other side; and (7) toe-off of the tested side and mid-stance of the other side. Three trials were performed and averaged for each subject. Seven pairs of fluoroscopic images were imported to Rhinoceros (McNeel & Associates, WA, USA) and the 3D ankle bone models were registered semi-automatically [19] by matching their projected shapes to the fluoroscopic images.

### 2.4. Coordinate System Definition and Model Alignment

The global coordinate system was defined based on the subject-specific bone geometry of the tibia and described by Yamaguchi et al. [20]. For all models, the tibial bones were aligned and the line connecting the medial–lateral and anteroposterior center points of the distal tibial shaft at 5 and 10 cm above the joint surface was defined as the superior–inferior axis (*z*-axis), and the origin was the crossing point of this axis and the tibial plafond. The anterior–posterior axis (*x*-axis) was defined as the line perpendicular to the line connecting the anteromedial and anterolateral edge of the tibial plafond. The medial–lateral axis (*y*-axis) was a cross product of the superior–inferior and anterior–posterior axes. Cartesian coordinate systems were established in 3-Matics (Materialise NV., Leuven, Belgium).

### 2.5. Defining the GATJ

**Figure 2:**
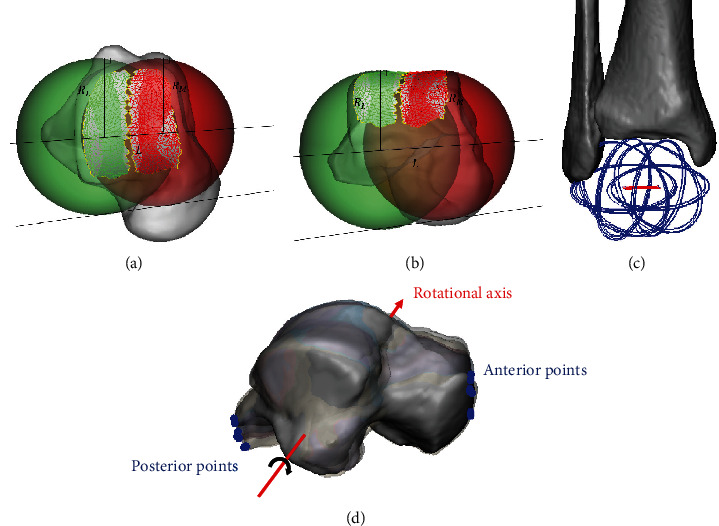
(a) Top view and (b) front view of the measurement of the parameters of fitting spheres (the radius of the lateral sphere *R*_L_, the radius of the medial sphere *R*_M_, the distance between sphere origins L), (c) fitting spheres and the geometric axes of each talus at all poses, (d) an illustration of the most anterior point at the navicular articular surface and the most posterior point at the posterior process of each talus at all poses.

We used a two-sphere-fitting method described in previous studies [21, 22] to find the geometric axes from the surface morphology of the talus. This method defined the axis by connecting the two centers of the least-squares fitting spheres to the medial or lateral part of the trochlear surface of the talus. The medial or lateral part of the trochlear surface of the talus was defined and manually selected as the facet surface between the central trochlea groove and the medial or lateral rim. A sensitivity analysis of the area of the selected trochlear surface of the talus in Figure S1 and Table S1 of the Supplementary File showed that changing the surface area made a small impact on the radius of the spheres and the position of the sphere origins (the maximum difference of the radii of the medial and lateral fitting spheres were both less than 5%). The line connecting the center points of the medial and lateral spheres was the GATJ. The middle point of GATJ was defined as the middle point between the center points of two spheres (the two-sphere-fitting method and geometric axes of talus at all poses were illustrated in Figure 2). The radius of the lateral sphere *R*_L_, the radius of the medial sphere *R*_M_, and the distance between sphere origins L were measured (Figures 2(a) and 2(b)). The relative distances from the middle point of each axis to the origin along to the *x*, *y*, and *z*-axis of the coordinate system of each model were measured, respectively, and labeled as “A–P distance”, “M–L distance”, and “S–I distance”. The angle deviations between each axis and the coronal, sagittal, or transverse plane of the coordinate system of each model were also quantified and labeled as “Angle to the coronal plane”, “Angle to the sagittal plane”, and “Angle to the transverse plane”. Data were processed in 3-Matics (Materialise NV, Leuven, Belgium).

### 2.6. Verification of the GATJ

To verify that the talus rotated about the GATJ during gait, we selected two feature points of the talus at each pose and calculated the distance from each point to the geometric axis of the talus. The most anterior point at the navicular articular surface of the talus and the most posterior point at the posterior process of the talus were used (Feature points of the talus were illustrated in Figure 2(d)). We calculated the shortest distance from points to the GATJ of the talus at pose 4. The calculation of the shortest distance between one point and one line was implemented in MATLAB (2018b, Mathworks Inc., Natick, MA).

### 2.7. Statistical Analysis

All data were presented as mean values with standard deviation. All data sets were checked for normal distribution and homogeneity of variance. A paired *t*-test was used to determine the significance of the difference between the radius of the medial and lateral sphere or between two poses. One-way repeated measures ANOVA was conducted to compare the distance or angles among each group. Statistical significance level *p* was set at 0.05. Data were processed in MATLAB (2018b, Mathworks Inc., Natick, MA).

## 3. Results

### 3.1. Measurement of Parameters of Fitting Spheres for Talar Trochlea

The radii of the medial and lateral fitting spheres of the 15 samples were all normally distributed and showed no significant difference in variance. The radius of the medial fitting sphere (*R*_M_) was 19.52 ± 1.45 mm, while the radius of the lateral fitting sphere (*R*_L_) was 20.61 ± 1.40 mm, *p* < 0.05. For all samples, *R*_L_ was larger than *R*_M_. The distance between sphere origins (L) was 15.22 ± 1.49 mm.

### 3.2. Measurement of Position and Orientation of the GATJ

**Figure 3:**
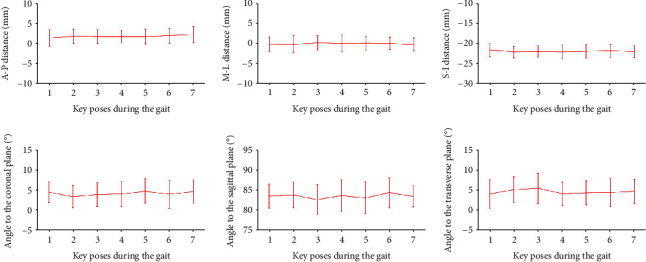
The position (A–P distance, M–L distance, S–I distance) and (Angle to the coronal, sagittal, and transverse plane) of the GATJ at seven key poses (A–P: Anterior–Posterior; M–L: Medial–Lateral; S–I: Superior–Inferior).

The position and orientation of the GATJ were presented in Figure 3 and listed in Table S2 of the Supplementary File. All data of the 15 samples were all normally distributed and showed no significant difference in variance. The averaged anterior–posterior, medial–lateral, and superior–inferior distances between the middle points of the GATJ to the coordinate origins were from 1.351 mm to 2.140 mm (*p* = 0.9534 > 0.05), from –0.27 mm to 0.11 mm (*p* = 0.9949 > 0.05), from –22.16 mm to –21.67 mm (*p* = 0.9784 > 0.05), respectively.

The averaged angles between the GATJ and coronal plane, sagittal plane, transverse plane were from 3.38 to 4.763 degrees (*p* = 0.8586 > 0.05), from 82.65 to 84.29 degrees (*p* = 0.8446 > 0.05), from 3.90 to 5.34 degrees (*p* = 0.8711 > 0.05), respectively. The overall angles between the GATJ and coronal plane, sagittal plane, transverse plane were 4.18±2.91 degrees, 83.24 ± 3.31 degrees, and 4.47 ± 3.20 degrees, respectively. The position and orientation of the GATJ showed no significant difference for all poses during gait.

### 3.3. Verification of the GATJ

**Figure 4:**
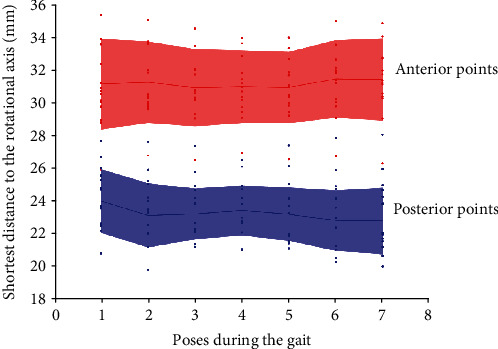
Shortest distance from the anterior or posterior points of the talus to the GATJ at the stance phase of the gait cycle (Pose 4).

The shortest distance from two feature points of the talus to the geometric axis of the talus at the stance phase of the gait cycle (pose 4) was presented in Figure 4 and listed in Table S3, SI. The average distances from the talar anterior point to the GATJ of the talus at pose 4 were between 30.89 and 31.42 mm, while that from the talar posterior point were between 22.75 and 23.97 mm. For both the anterior and posterior points of seven key poses, their distance to the GATJ of the talus at pose 4 both showed no significant difference during gait (*p* = 0.9973 > 0.05 for the anterior points and *p* = 0.774 > 0.05 for the posterior points).

## 4. Discussion

In this study, we utilized talar morphology to define the GATJ by sphere-fitting the medial and lateral part of the trochlear surface of the talus and measured the orientation and position of this axis in vivo. The findings of the current study found no statistically significant difference in the shortest distance of feature points of the talus to the geometric axis and the position and orientation of the GATJ among seven key poses of the gait cycle, indicating the GATJ was the axis about which the talus rotated and it was one fixed axis during gait. Although Parr et al. [21] first introduced this method to define the GATJ and Nichols et al. [22] used this method to predict the tibiotalar joint motion in inverse kinematic models, to our knowledge, it is the first study to in vivo evaluate the position and orientation of the GATJ defined by the surface morphology of the talus.

Generally, the rotational axis of the tibiotalar joint was considered as an obliquely transverse axis passing close to the malleoli. Pioneer work by Inman [2] measured the shape and rotational motion of the talus on cadaveric specimens and concluded that the shape of the talar trochlea can be fitted as a truncated cone which had a mean angle of 82.7° between the axis and the midline of the tibia, which was 7.3° between the axis and the transverse plane. This concept of one fixed axis was questioned by both in vitro cadaver studies and in vivo studies, but their results of the measurement of the rotational axis of the tibiotalar joint were inconsistent [8, 10, 14, 23]. The orientations of the rotational axes measured from previously published data are summarized in Table 1. Our findings supported the concept of one fixed axis of the tibiotalar joint and our data of the orientation of the GATJ were in the range of reported data. Possible causes of the discrepancy of the results included different sample populations and measurement methods. There also existed a discrepancy in reporting the orientation, some researchers preferred to calculate the angle to the coordinate planes, while others tended to report the angle after the projection in one of the coordinate planes [7, 13]. These discrepancies cannot be ignored and should be clarified in future studies.

With the increased use of total ankle replacement for the treatment of end-stage ankle arthritis in recent years, researchers and manufacturers are making efforts to perfect the design of total ankle implants, thus improving their performance. A novel implant with its rotational axis same as that of the tibiotalar joint can better replicate the natural motion of the ankle. Our findings in this study encouraged us to propose a design process of the talar component of the total ankle implant. The surface of the talar trochlea can be fitted by two spheres and the rotational axis is the line connecting the sphere origins about which the talus should rotate during gait. The radii of the fitting spheres were in the range of the previously reported values [4, 5, 24–28]. Moreover, the sphere-fitting method to design the talar component of the total ankle implant provided a central sulcus in the implant, thus increasing more medial–lateral stability, compared with the truncated cone fitting method proposed by Inman [2].

The current study has some limitations. First, a small number of subjects was used. Although all data presented a normal distribution and no significant difference of variance, more subjects should be included in future studies to increase the credibility of the current findings. Furthermore, only one method of defining the rotational axis of the tibiotalar joint was used in this study. The advantages and disadvantages of different defining methods were distinct. Future studies should evaluate the difference in the position and orientation of the axes derived from various methods. Last, the current data of radius were not taken the cartilage layer into account due to the fact that all parameters were measured in CT images. Therefore, the radius data for the design of the talar component of the total ankle implants should add the thickness of the articular cartilage layer of the trochlea of the talus, which was reported as averaging 1.35 mm in males and 1.11 mm in females [29]. Since the articular cartilage distributes uniformly on the trochlea of the talus, not including the cartilage layer should put a small influence on the location of the fitting sphere origins [29]. The current proposal of the design process of the talar component of the total ankle implant has not been validated. An implant manufactured based on this design process should be tested in future studies with biomechanical experiments and, if possible, clinical trials to prove the superiority of this design.

## 5. Conclusion

This study presented the in vivo measurement of the GATJ defined by the morphology of the talar trochlea. This geometric axis was proved to be the axis about which the talus rotated and it was one fixed axis during gait. The current finding may help the design of the talar component of the total ankle implant.

## Figures and Tables

**Table 1:** A summary of the orientation of the rotational axis measured from previously published data.

Author + published year	Sample or subject size	Data type	Angle to the coronal plane (°)	Angle to the sagittal plane (°)	Angle to the transverse plane (°)
Inman [2]	107	Direct measurements of cadaver tali	—	—	7.3 ± 3.6
Arndt et al. [14]	3	In vivo kinematic data	—	54.3 to 73.8	7.5 to 33.6
Lewis et al. [8]	3	Kinematic data of cadaver samples	18.5 to 24.7	—	–3.4 to 2.4
Sheehan [10]	25	Dynamic MRI images	15.8 ± 12.2	68 ± 41.7	5.5 ± 12.9
Claassen et al. [23]	98	CT images of cadaver samples	10.84 ± 7.10	104.5 ± 6.51	3.78 ± 8.13
Current study	15	CT images + in vivo kinematic data	4.18±2.91	83.24 ± 3.31	4.47 ± 3.20

## Data Availability

All data used to support the findings of this study are available from the corresponding author upon request.

## Supplementary Materials

Supplementary Materials The Supplementary File in the present study included sensitivity analysis of the selection on the surface of talar trochlea for sphere-fitting, values of the position and orientation of the GRATJ at seven key poses, and values of the shortest distances from the anterior or posterior points of the talus to the GRATJ at the stance phase of the gait (Pose 4). Figure S1, Tables S1, and S2 of the Supplementary File were referred to in the manuscript.
