# Supplementary material for: In Vivo Evaluation of the Position and Orientation of the Geometric Axis of the Tibiotalar Joint
Source: Appl Bionics Biomech. 2023 Jan 18;2023:2763099. doi: 10.1155/2023/2763099 (PMC9876701; doi:10.1155/2023/2763099)
Supplement: Supplementary Materials — The Supplementary File in the present study included sensitivity analysis of the selection on the surface of talar trochlea for sphere-fitting, values of the position and orientation of the GRATJ at seven key poses, and values of the shortest distances from the anterior or posterior points of the talus to the GRATJ at the stance phase of the gait (Pose 4). Figure S1, Tables S1, and S2 of the Supplementary File were referred to in the manuscript. [file 2763099.f1.pdf]

Supplementary File

1. Sensitivity analysis of the selection on the surface of talar trochlea for sphere fitting

Before defining the geometric axis of the talocrural joint for all subjects, a sensitivity analysis of the selection on the surface of talar trochlea for sphere fitting on one of the subjects was performed. First, manually selected the facet surface between the central trochlea groove and the medial or lateral rim. Then, shrink the selection by one mesh from the edge to the center. Last, sphere fitting each selection. (The sensitivity analysis was illustrated in **Figure S1**）

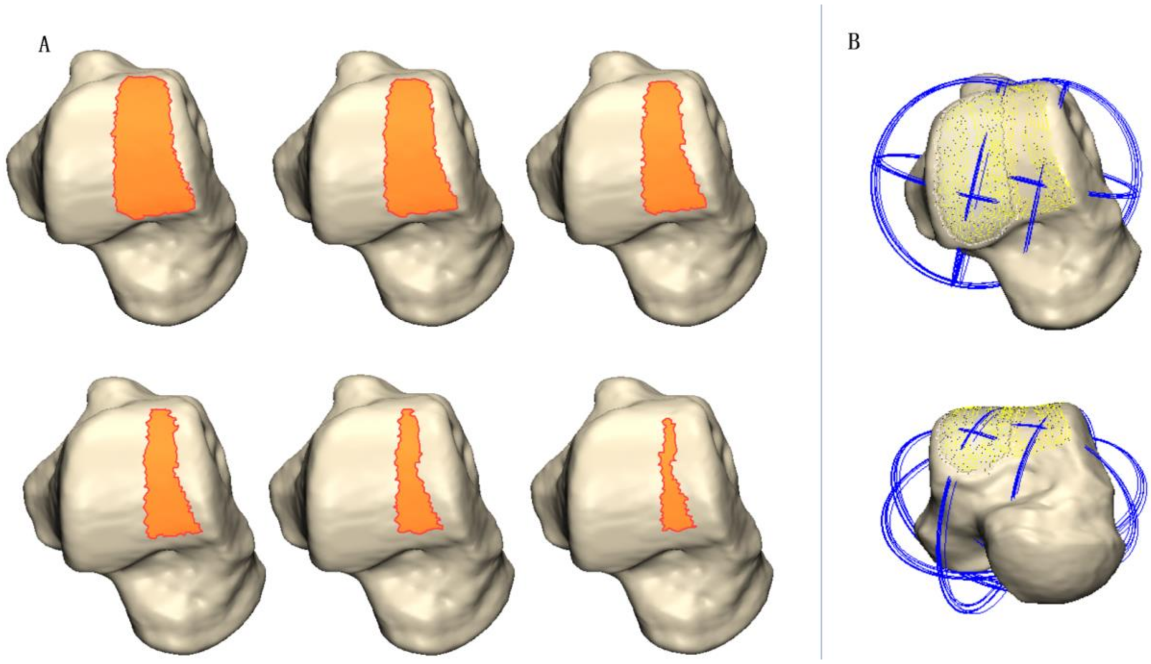

Figure S1 An illustration of the sensitivity analysis of the area selection on the surface of talar trochlea for sphere fitting. (A) Shrinking the area of selection (Take medial facet of the talar trochlea as an example); (B) Sphere fitting each area selected.

The result for the sensitivity analysis was listed in **Table S1**. The positions of the sphere origins and the radii of the medial or lateral fitting sphere were not sensitive to the selection area. The maximum difference among different areas selected for medial and lateral spheres (from 87.64mm^2 to 441mm^2 and from 59.68mm^2 to 553.00mm^2, respectively) were 4.04% and 4.39%, respectively, which were both less than 5%.

Table S1 Sensitivity analysis of the selection on the surface of talar trochlea for sphere fitting.

	Area (mm^2)	Number of meshes	Number of nodes	Radius (mm)	Coordinates of sphere origins
Medial Surface 1	441.00	1594	869	20.59	111.65，151.10，267.24
Medial Surface 2	363.75	1327	728	20.03	111.36，151.54，267.26
Medial Surface 3	289.20	1076	600	19.84	111.22，151.62，267.14
Medial Surface 4	218.01	822	475	20.73	110.53，150.65，266.71
Medial Surface 5	149.03	567	340	19.98	110.97，151.92，267.33

Medial Surface 6	87.64	344	222	19.82	110.86，151.36，266.58
The maximum difference of the radii of the medial fitting spheres：				4.39%	
Lateral Surface 1	553.00	1877	1011	22.10	117.24，149.58，280.89
Lateral Surface 2	472.07	1599	868	22.90	117.64，148.68，281.14
Lateral Surface 3	390.27	1311	729	22.99	117.71，148.52，281.28
Lateral Surface 4	310.23	1037	583	22.11	117.40，149.34，281.40
Lateral Surface 5	236.61	794	455	22.86	117.60，148.49，281.33
Lateral Surface 6	170.62	579	340	22.16	117.28，149.12，281.20
Lateral Surface 7	112.47	382	240	22.66	117.25，148.54，280.81
Lateral Surface 8	59.68	202	143	23.03	116.89，148.12，279.56
The maximum difference of the radii of the lateral fitting spheres：				4.04%	

2. The position and orientation of the GRATJ at seven key poses

Table S2 The position (A-P distance, M-L distance, S-I distance) and orientation (Angle to the coronal, sagittal, and transverse plane) of the GRATJ at seven key poses (A-P: Anterior-Posterior; M-L: Medial-Lateral; S-I: Superior-Inferior)

	Pose 1	Pose 2	Pose 3	Pose 4	Pose 5	Pose 6	Pose 7
A-P distance (mm)	1.351± 1.994	1.733± 1.735	1.677± 1.684	1.711± 1.545	1.702± 1.754	1.876± 1.796	2.140± 2.000
M-L distance (mm)	-0.266 ± 1.747	-0.206 ±2.064	0.105± 1.715	-0.030± 1.999	0.044± 1.606	-0.047± 1.433	-0.265± 1.548
S-I distance (mm)	-21.671 ± 1.616	-22.164 ±1.423	-22.042 ±1.427	-22.146 ±1.704	-22.015 ±1.718	-21.845 ±1.598	-22.077 ±1.446
Angle to the coronal plane (°)	4.534 ± 2.551	3.379 ± 2.762	3.887 ± 2.933	4.072 ± 3.062	4.763 ±3.052	3.971 ±3.471	4.680 ±2.791
Angle to the sagittal plane (°)	83.439 ± 2.845	83.747± 3.122	82.648± 3.630	83.547± 3.892	83.006± 3.926	84.286± 3.624	83.380± 2.527
Angle to the transverse plane (°)	3.896 ± 3.521	5.002± 3.177	5.340± 3.725	3.971± 2.893	4.196± 2.967	4.284± 3.454	4.583± 2.982

3. Verification of the GRATJ

To verify that the talus rotated about the GRATJ during the gait, we selected two feature points (The most anterior point at the navicular articular surface of the talus and the most posterior point at the posterior process of the talus) of the talus of each pose and calculated the distance from each point to the rotational axes of the talus (See **Table S3**).

Table S3 Shortest distance from the anterior or posterior points of the talus to the GRATJ at the stance phase of the gait (Pose 4).

Distance (mm)	Pose 1	Pose 2	Pose 3	Pose 4	Pose 5	Pose 6	Pose 7
Anterior points	31.1076 ±2.7591	31.2139 ±2.4714	30.8853 ±2.3491	30.9459 ±2.2109	30.8997 ±2.1642	31.4201 ±2.3521	31.3905 ±2.5002
Posterior points	23.9748 ±1.9346	23.1035 ±1.9444	23.1921 ±1.5386	23.4041 ±1.5159	23.1744 ±1.6150	22.7846 ±1.8248	22.7534 ±2.0204
